# Serum C-reactive protein concentrations in Nova Scotia Duck Tolling Retrievers with immune-mediated rheumatic disease

**DOI:** 10.1186/s13028-017-0289-9

**Published:** 2017-04-17

**Authors:** Hanna Dorotea Bremer, Anna Hillström, Malin Kånåhols, Ragnvi Hagman, Helene Hansson-Hamlin

**Affiliations:** 1 Department of Clinical Sciences, Faculty of Veterinary Medicine and Animal Science, Swedish University of Agricultural Sciences, Box 7054, 750 07 Uppsala, Sweden; 2 Clinical Pathology Laboratory, University Animal Hospital, Swedish University of Agricultural Sciences, Uppsala, Sweden

**Keywords:** C-reactive protein, Canine, Immune-mediated rheumatic disease, Nova Scotia Duck Tolling Retriever, Systemic lupus erythematosus

## Abstract

Nova Scotia Duck Tolling Retrievers (NSDTRs) are a dog breed often affected by immune-mediated rheumatic disease (IMRD), a disorder characterised by chronic stiffness and joint pain. Most, but not all, dogs with IMRD, have antinuclear antibodies (ANA), which are also commonly present in the autoimmune disease systemic lupus erythematosus (SLE). The clinical and diagnostic findings of IMRD indicate that it is an SLE-related disorder. C-reactive protein (CRP), an acute phase protein, is a quantitative marker of inflammation for many diseases and is used for diagnosing and monitoring systemic inflammation in both humans and dogs. However, in human SLE, CRP concentrations are often elevated but correlate poorly with disease activity; they can be low in individual patients with active disease. The aim of the study was to investigate CRP in a group of NSDTRs with the SLE-related disorder IMRD. The hypothesis was that CRP concentrations would be increased in dogs with IMRD compared to healthy dogs, but that the increase would be mild. Serum CRP concentrations were measured in 18 IMRD-affected NSDTRs and 19 healthy control NSDTRs using two different canine-specific CRP assays. Dogs with IMRD and ANA had higher CRP concentrations than the control dogs, but the concentrations were below the clinical decision limit for systemic inflammation for most of the IMRD dogs. These results indicate that CRP concentrations were increased in dogs with IMRD and ANA, but the increase was mild, similar to what has been observed in human SLE.

## Findings

Nova Scotia Duck Tolling Retriever (NSDTR) dogs are, compared to other dog breeds, at increased risk of autoimmune disorders and particularly immune-mediated rheumatic disease (IMRD) [1, 2]. Dogs affected by IMRD have a clinical picture indicative of chronic non-erosive polyarthritis with stiffness and pain from multiple joints, sometimes in conjunction with other clinical manifestations. Most, but not all, IMRD cases display antinuclear antibodies (ANA) on indirect immunofluorescence (IIF) [2]. Antinuclear antibodies and non-erosive polyarthritis are also commonly present in the systemic autoimmune inflammatory disease, systemic lupus erythematosus (SLE), in humans as well as in dogs [3–5] and IMRD is considered an SLE-related disorder [2, 6].

Measurement of C-reactive protein (CRP), an acute phase protein produced in the liver during systemic inflammation, is used for diagnosing and monitoring inflammatory diseases in both humans and dogs [7–9]. CRP is a quantitative marker of inflammation [10], and its measurement is used to monitor disease activity in dogs with immune-mediated polyarthritis [11, 12]. Despite being a reliable marker of systemic inflammation, CRP concentrations in human SLE correlate inconsistently with disease activity. CRP concentrations are often elevated, but individual patients with active disease can have low CRP concentrations [13, 14]. In the present study, we investigated the CRP concentrations in a group of NSDTRs with the SLE-related disorder IMRD. The hypothesis was that the serum CRP concentrations in these dogs would be higher than in the healthy controls, but that the elevation would be milder than in immune-mediated polyarthritis in other breeds [11, 15, 16].

Serum CRP concentrations were measured in healthy and IMRD-affected NSDTRs. Serum samples from IMRD dogs had been collected in connection with other studies [2, 17, 18] and previously tested for the presence of ANA with IIF [19]. Samples were considered positive at a titre of ≥1:100. The study was approved by the Uppsala Animal Ethical Committee and Swedish Board of Agriculture (C417/12). In total, sera from 37 NSDTRs were included of which 18 had signs of IMRD and 19 were healthy controls (Table 1). Of the 18 sera from dogs with IMRD, nine were positive (ANA^pos^) and nine were negative (ANA^neg^) for the IIF-ANA test. All IMRD dogs had displayed clinical signs consistent with IMRD upon physical examination, including lameness or pain from two or more joints on manipulation [2]. The signs had been apparent for at least 14 days before sampling and no other causes than IMRD were suspected. None of the dogs had been treated with anti-inflammatory drugs at the time of sampling. The control dogs were considered healthy, based on normal physical examination and owner interview at the time of sampling and during a followup interview after 4 weeks. All the blood samples were collected by venipuncture into tubes with clot activator, after written owner consent. The blood samples from the IMRD dogs were either sent by post, or collected at, the University Animal Hospital, Swedish University of Agricultural Sciences, Uppsala, Sweden, where the samples were stored and analysed. The sera had been stored at −80 °C for a maximum of 8 years before the CRP analysis in the current study. Some of the samples had undergone up to four freeze–thaw cycles. The samples from control dogs had been collected at the University Animal Hospital and stored at −80 °C for a maximum of 3 months before analysis.

**Table 1:** Age, sex and ANA test result for the 37 dogs included in the study

Number	Group	Median age in years (range)	Female/male	ANA test result
9	IMRD	5 (3–8)	8/1	Positive
9	IMRD	7 (2–9)	3/6	Negative
19	Control	4 (1–8)	10/9	Negative

*ANA* antinuclear antibodies, *IMRD* immune-mediated rheumatic disease, *Control* healthy control dogs

For the current study, the serum samples were thawed and the serum CRP was measured in duplicate with a validated canine-specific immunoturbidimetric assay (Gentian cCRP, Gentian AS, Moss, Norway) [20], in a single run. For samples with CRP concentrations <6.8 mg/L, i.e., the assay’s limit of quantification (LOQ), re-analysis was performed in duplicate with a second, high-sensitivity canine-specific CRP assay (Gentian hsCRP) [21] on the same occasion. Analyses were performed on a fully automated, open-system clinical chemistry/immunoassay analyser (Abbott Architect c4000, Abbott Park, IL, USA). Statistical analyses were performed using software R V3.0.2. To compare the CRP concentrations between the three groups, Welch ANOVA was performed followed by pair-wise comparisons with Welch two-sample t test. Calculations were performed on log-transformed data. The significance level was set to P < 0.017 using Bonferroni adjustment for multiple testing. CRP concentrations <0.50 mg/L, corresponding to the LOQ of the hsCRP assay, were assigned the value 0.25 mg/L in the statistical analyses.

**Fig. 1:**
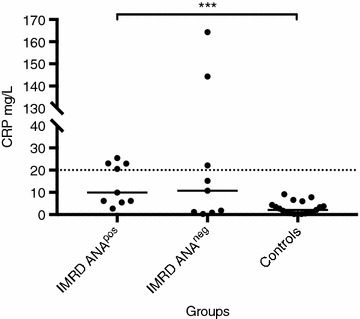
Serum concentrations of C-reactive protein (CRP) in Nova Scotia Duck Tolling Retriever (NSDTR) dogs with immune-mediated rheumatic disease (IMRD) and healthy controls. Serum CRP was measured with two different validated canine-specific assay (Gentian AS, Moss, Norway), values <6.8 mg/L correspond to concentrations measured with a high-sensitive assay. The horizontal dotted line represents CRP concentration 20 mg/L, the clinical decision limit for systemic inflammation. Observe the scale break on the y-axis. IMRD ANA^pos^, NSDTRs with IMRD and a positive indirect immunofluorescence antinuclear antibody (IIF-ANA) test; IMRD ANA^neg^, NSDTRs with IMRD and a negative IIF-ANA test. ***Significant difference (P < 0.001) between groups

Dogs with IMRD and ANA had higher CRP concentrations than the healthy control dogs (P < 0.001), but no significant difference was observed between the IMRD ANA^neg^ and control dogs. The CRP concentrations in individual dogs, in each of the three groups, are presented in Fig. 1. The median CRP concentration and range were 9.9 (2.7–25.4) mg/L in the IMRD ANA^pos^ dogs, 10.8 (<0.5–164.3) mg/L in the IMRD ANA^neg^ dogs and 2.1 (<0.5–9.2) mg/L in the control dogs.

Despite the statistically significant difference in CRP concentration between the IMRD ANA^pos^ and healthy dogs, several of the IMRD dogs in both the ANA^pos^ and the ANA^neg^ groups had concentrations similar to those of the healthy dogs. This resembles the CRP alterations in human SLE, where the CRP concentrations are often mildly elevated, but can also be low in individual patients with active disease [13]. In most of the IMRD dogs, the increase in CRP concentration was less than in immune-mediated polyarthritis in other dog breeds [11, 15, 16], but higher than concentrations reported for e.g., canine osteoarthritis [22]. A CRP of 20 mg/L has previously been established at our laboratory as the decision limit for clinical diagnosis of systemic inflammation. This decision limit was determined by performing an ROC curve analysis of CRP data from dogs with and without known systemic inflammatory disease (data not shown), similarly to what has been previously described [23]. In the present study, most of the IMRD dogs had CRP concentrations below or just above the clinical decision limit. Measurement of CRP concentrations below the clinical decision limit is necessary to detect smaller changes between healthy and diseased dogs, as shown in the present study.

There was no significant difference between the IMRD ANA^neg^ dogs and healthy controls, but two dogs had markedly elevated CRP concentrations (>100 mg/L). These two had typical clinical signs of IMRD, but the diagnosis of IMRD can be challenging, especially in the absence of ANA. It is also possible that these dogs suffered from disorders other than, or in addition to, IMRD that induced systemic inflammation, with resulting high CRP concentrations. The CRP concentrations in these two dogs were of similar magnitude as those reported in other studies of immune-meditated polyarthritis [11, 15, 16]. In a study by Hillström et al. [16], CRP was measured with the same assay as here and the median CRP concentration in dogs with immune-mediated arthritis was 118.0 mg/L, which is considerably higher than the medians in the present study. Unfortunately, direct comparison of CRP concentrations between other studies is not feasible because of the lack of standardization of CRP measurements.

Different veterinarians examined the dogs, which is a limitation of the study. Another limitation is that handling and storage of the samples were not standardised. However, CRP is stable at room temperature [20], and in freezer storage [24] and is unaffected by repeated freeze–thaw cycles [20]. The results of the present study indicate that CRP concentrations increased in NSDTRs with IMRD, but the concentrations varied and were often below the clinical decision limit for systemic inflammation. Two of the individual IMRD ANA^neg^ dogs had high CRP concentrations which might indicate that they suffered from concurrent or disorders other than IMRD. The CRP alterations in IMRD ANA^pos^ dogs are similar to those reported in humans with SLE [13]. Whether CRP has a potential clinical value for diagnosing and monitoring IMRD in individual dogs, warrants further investigations.

## Abbreviations

ANA: antinuclear antibodies
ANA^pos^: antinuclear antibody positive
ANA^neg^: antinuclear antibody negative
CRP: C-reactive protein
hsCRP: high-sensitivity CRP
IIF: indirect immunofluorescence
IMRD: immune-mediated rheumatic disease
LOQ: limit of quantification
NSDTR: Nova Scotia Duck Tolling Retriever
SLE: systemic lupus erythematosus
